# A refractory case of *CDK4*-amplified spinal astrocytoma achieving complete response upon treatment with a Palbociclib-based regimen:a case report

**DOI:** 10.1186/s12885-020-07061-3

**Published:** 2020-07-08

**Authors:** Jietao Lin, Ling Yu, Yuanfeng Fu, Hanrui Chen, Xinting Zheng, Shutang Wang, Chan Gao, Yang Cao, Lizhu Lin

**Affiliations:** 1 Oncology Center, the First Affiliated Hospital of Guangzhou University of Chinese Medicine, 16th Airport Road, Guangzhou, 510405 Guangdong China; 2 Guangzhou University of Chinese Medicine, 12th Airport Road, Guangzhou, 510405 Guangdong China; 3 Shanghai Institute of Cardiovascular Diseases, Zhongshan Hospital, Fudan University, 180 Fenglin Road, Shanghai, 200032 China; 4 Medical Affairs,3D Medicines Inc., Building 2, Block B, 158 XinJunhuan Street, Pujiang Hi-tech Park, MinHang District, Shanghai, 201114 China

**Keywords:** Spinal astrocytoma, next-generation sequencing, Palbociclib, Targeted therapy

## Abstract

**Background:**

Spinal cord astrocytoma is a rare neoplasm, and patients usually recur within months after surgery. There is currently a lack of consensus regarding post-operative treatment. Clinical data on the activity of systemic treatment like chemoradiotherapy and anti-angiogenic agents also remained scant. Next-generation sequencing (NGS) -based genomic profiling thus may help identify potential treatment options for a subset of patients that harbor actionable genetic alterations.

**Case presentation:**

We reported for the first time a refractory case of grade III spinal cord astrocytoma that underwent two surgeries but eventually progressed following post-operative chemoradiotherapy plus bevacizumab. Hybridization capture-based NGS using a 381-gene panel disclosed cyclin dependent kinase 4 (*CDK4*) amplification and after receiving a triplet regimen containg palbociclib for 15 months, the patient achieved complete response.

**Conclusions:**

This case demonstrated the importance of genetic profiling and the benefit of a multi-modality treatment strategy in cancer management.

## Background

Astrocytomas are a rare group of glial neoplasms of the central nervous system (CNS). They arise from astrocytes, supporting cells of the nervous system, and only 3% of astrocytomas are found in the spinal cord [1]. Spinal cord astrocytoma (SCA) comprises 2.1% of all adult primary spinal cord tumors, which in turn, accounts for 2–4% of all CNS tumors [2, 3]. The prognosis of SCA patients depends on the tumor grade (grade I-IV according to World Health Organization criteria) and duration of symptoms before diagnosis, where high-grade ones are usually highly aggressive and may cause neurological deficiency or even death [4]. There are currently limited treatment options available for SCAs. Surgery serves as the initial treatment modality; however, complete resection is often not possible due to the infiltrative nature of astrocytoma [1, 4]. Although post-operative spinal radiation has been adopted worldwide to prevent recurrence, its exact role in SCA management remained controversial because low-grade SCAs may benefit minimally from radiotherapy due to low spontaneous recurrence rates while high-grade SCAs generally have low sensitivity to radiation [1, 4]. Likewise, established chemotherapy regimens such as temozolomide, administered alone or in combination with bevacizumab, are also considered to have limited value in treating SCAs since they have not been systematically examined or validated in large prospective studies [4]. Multi-modality therapy is, therefore, of paramount importance in such a scenario and next-generation sequencing (NGS)-guided targeted therapy may serve as a last resort for certain patients. We herein reported a *CDK4*-amplified case of SCA achieving complete response following multi-modality therapy containing palbociclib.

## Case presentation

**Fig. 1:**
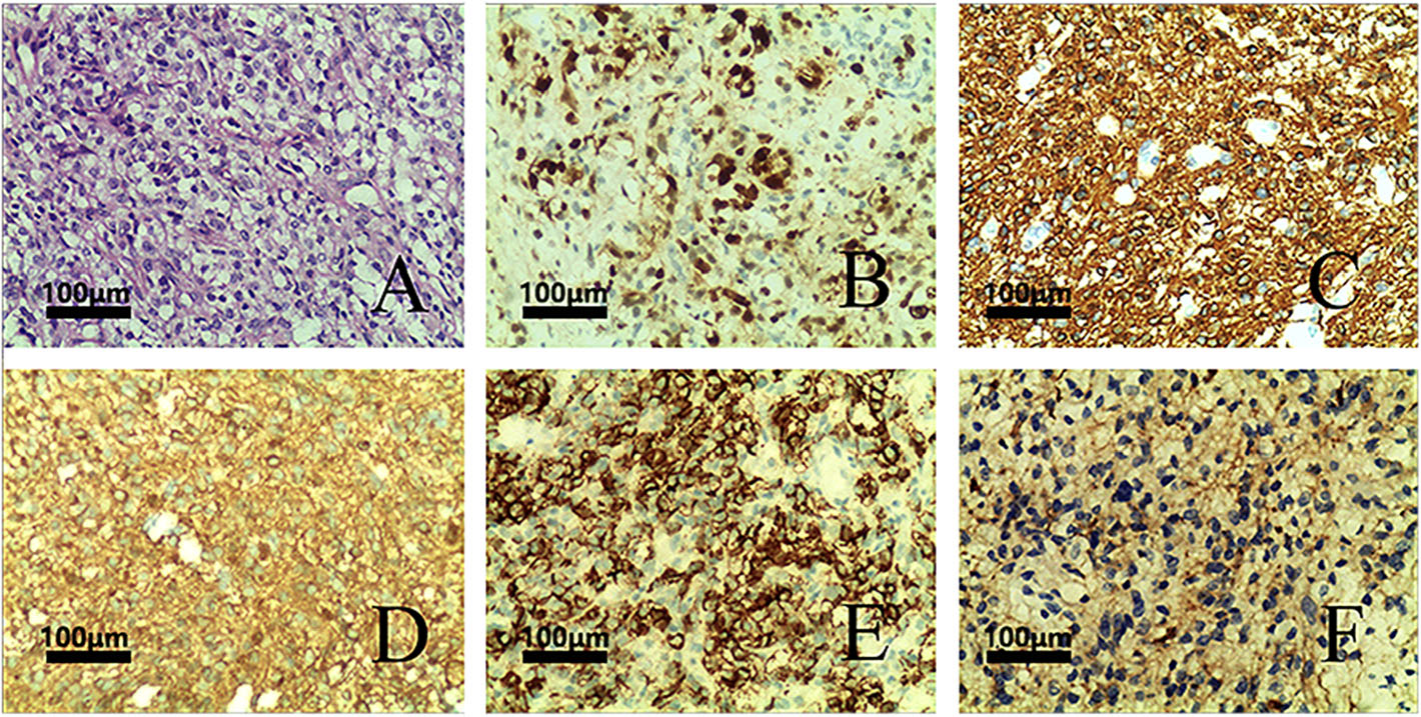
Histologic features of the tumor. **a** H&E section showing diffuse invasion of tumor cells with abundant cytoplasm, indicative of anaplastic oligodendrocytic astrocytoma, WHO III grade; **b**) IHC showing KI-67: 70% (+); **c**) IHC showing focal staining of GFAP focal; (+) **d**) IHC showing strong diffuse staining of Vimentin (+); **e**) IHC showing CD56 partly (+); **f**) IHC showing Syn partly (+). Original magnifications: **a**-**f**:200×. H&E:haemotoxylin and eosin. IHC: immunohistochemistry

A 38-year-old man with a decade’s history of chronic hepatitis B virus infection presented with lower back pain in March, 2016. He did not have any hereditary diseases, a family history of cancer, a history of trauma, or any other chronic medical conditions. Spinal magnetic resonance imaging (MRI) disclosed a mass measuring 10 × 14 mm in the 10th thoracic segment of his spinal cord on March 31st. The tumor was surgically removed on April 13th, 2016. Post-surgical pathology revealed anaplastic astrocytoma (WHO grade III). Immunohistochemical staining demonstrated that the tumor was positive for Vimentin (+++), GFAP (+), S-100 (+), Syn (focally +), Ki-67(70% +), and p53 (partly +), but negative for CgA and EMA (Fig. 1, Supplemental Table 1). An Olympus BX41 microscope with a 10× ocular lens and a 20× objective lens was used for microscopy and an MShot MD3 microscope camera along with Mshot Image Analysis System was used for image acquisition. The images were acquired at a resolution of 96 dpi and Adobe Photoshop was used to enhance the resolution of the images to 300 dpi. Both H3.3 histone A (H3F3A) and histone cluster 1, H3b (HIST1H3B), which are commonly mutated in pediatric midline glioma and sometimes in adult patients, were shown to be wild-type using fluorescence in situ hybridization (FISH). The patient did not harbor any dehydrogenase (*IDH*) mutations or 1p/19q co-deletion, either according to FISH.

**Fig. 2:**
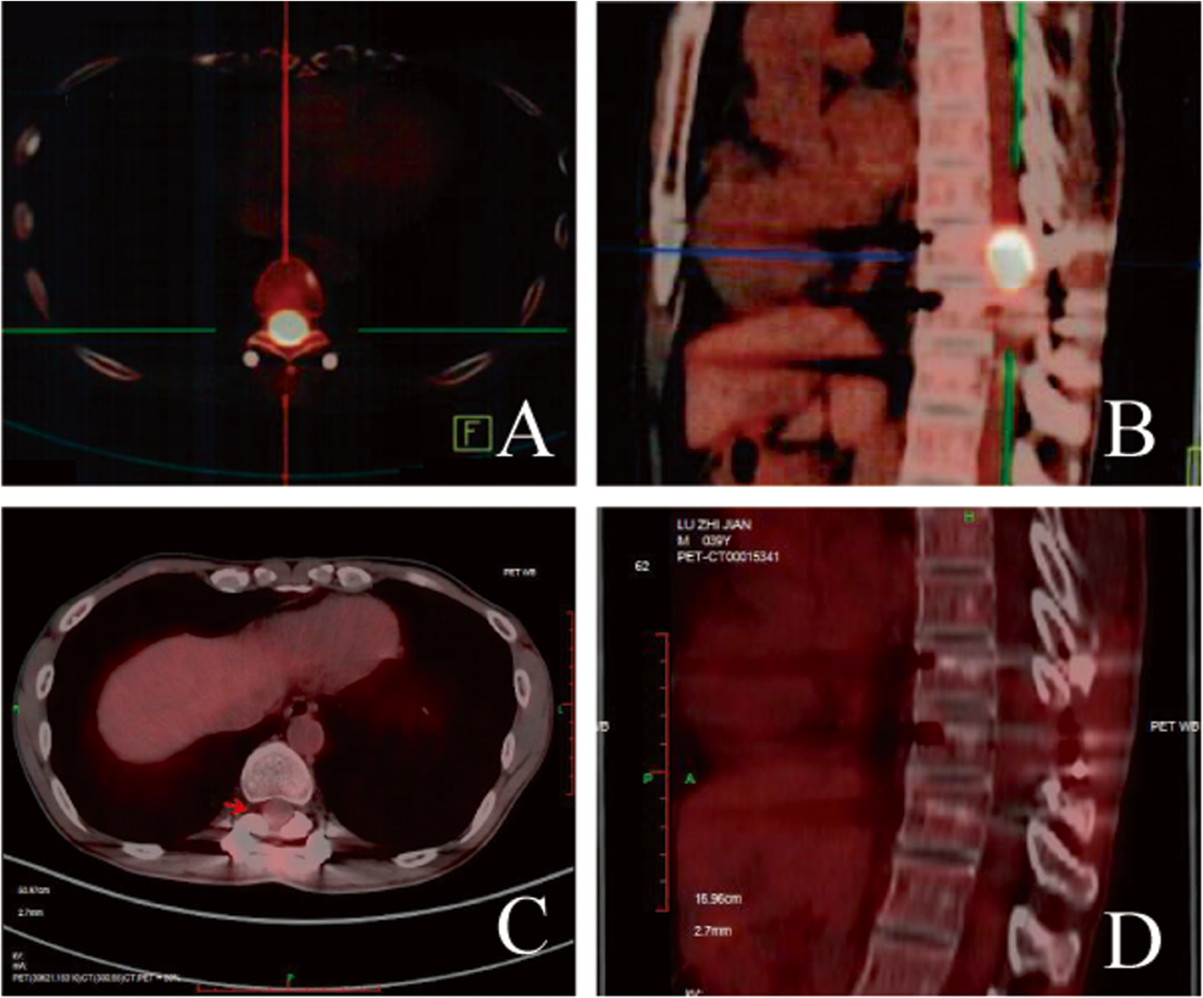
Positron emission tomography-computed tomography (PET-CT) scans showing the second recurrence in the 10th thoracic spinal cord on April 17th, 2017 (**a** and **b**), and complete response on August 24th, 2018 (**c** and **d**)

On September 18th, 2016, a follow-up MRI scan revealed local recurrence of the primary lesion, and a second surgical excision was performed on September 26th, 2016. Histological examination confirmed the initial pathological diagnosis of anaplastic astrocytoma. Following surgery, adjuvant chemotherapy consisting of 4 cycles of nedaplatin (50 mg ivgtt D1-D3) and temozolomide (250 mg po D1-D5) was administered every 28 days. In March, 2017, the patient experienced an onset of progressive numbness and weakness in the lower limbs. The dysesthesias and weakness in the lower limbs became intensified later in April. Positron emission tomography-computed tomography (PET-CT) showed a hypermetabolic lesion in the 10th thoracic spinal cordon April 17th, 2017 (Fig. 2.A.). From April 24th, 2017 to April 29th, 2017, the patient underwent gamma knife radiosurgery at a marginal dose of 40 Gy and this was followed by four cycles of chemotherapy comprising bevacizumab (500 mg ivgtt D1), irinotecan (190 mg ivgtt D1) and temozolomide (250 mg po D1-D5) administered every 28 days.

The adjuvant chemoradiotherapy failed to control disease progression as a CT scan conducted on July 20th, 2017 indicated a second recurrence. Resected tissue sample obtained during the second surgery was therefore subjected to NGS analysis using a 381-gene panel (3DMedicine Clinical Laboratory, China) (Supplemental Table 2). As summarized in Table 1, genetic alterations identified included amplification of the genes encoding cyclin dependent kinase 4 (*CDK4*), murine double minute-2 (MDM2), fibroblast growth factor receptor substrate 2 (*FRS2*)*,* and GLI family zinc finger 1 (*GLI1*) and point mutations in WEE1 G2 checkpoint kinase (*WEE1*, c. 1385–1 G > A) and protein tyrosine phosphatase non-receptor type 11 (*PTPN11*, p.E69K). The patient was also found to be microsatellite stable (MSS) and hence was not likely to benefit from immunotherapy. Taken together, among all the genetic abberations identified, *CDK4* amplifcation was the only one that was potentially targetable. Amplification of *CDK4* may result in dysregulation of the cycline-D- cyclin-dependent kinase 4/6 (*CDK4/6*)-INK4-Rb pathway and eventually cause cell cycle progression and tumorigenesis [5]. Palbociclib, a selective oral inhibitor of *CDK4/CDK6*, binds to the ATP pockets of CDK4/6 and leads to cell cycle arrest at G1 phase [6]. Although palbociclib had not been approved for treating CNS malignancies, in a phase II study conducted on 30 patients diagnosed with *CDK4*-amplified advanced well-differentiated or dedifferentiated liposarcoma (WD/DDLS), palbociclib generated an estimated 12-week progression free survival (PFS) rate of 66%, well exceeding the pre-specified 40% 3-month PFS rate to consider the study positive [7]. The patient was, therefore, started on four cycles of palbociclib (125 mg po d1–21 q4w) plus temozolomide (250 mg po d1–5 q4w) on September 10th, 2017. MRI scans conducted every 2 months showed continuous tumor regression, and the symptoms also became stable with the sensation in the lower limbs gradually alleviated. Temozolomide was discontinued on March 28th, 2018 due to intolerable myelosuppression while palbociclib was continued for another two months before temozolomide was resumed along with apatinib when the patient’s conditions improved. Apatinib, in combination with chemotherapy, has been shown to be both effective and tolerable in adult patients with recurrent glioma and was hence included in the treatment regimen [8]. The triplet regimen lasted for two months, and a PET-CT scan conducted on August 24th, 2018 showed complete response (Fig. 2.B.). Apatinib was discontinued on September 15th, 2018, and the patient stopped taking temozolomide and palbociclib on April 20th, 2019. The patient was alive till the last follow-up on August 15th, 2019.

**Table 1:** Gene mutational profile of the spinal astrocytoma patient by next-generation sequencing

	mutation	Mutation abundance (%)/copy number
*CDK4*	amplification	32
*WEE1*	c.1385-1G > A	38.30%
*MDM2*	amplification	37
*PTPN11*	p.E69K	31.30%
*FRS2*	amplification	37
*GLI1*	amplification	32

Abbreviations: CDK4 = cyclin dependent kinase 4, WEE1 = WEE1 G2 checkpoint kinase, MDM2 =  MDM2 proto-oncogene, PTPN11 = protein tyrosine phosphatase non-receptor type 11, FRS2 = fibroblast growth factor receptor substrate 2, GLI1 = GLI family zinc finger 1

## Discussion and conclusions

Spinal astrocytomas are rare intramedullary CNS tumors, and evidence regarding efficacious systemic therapeutic agents is too scant to inform specific recommendations according to the National Comprehensive Cancer Network (NCCN) guidelines for central nervous system cancers [9]. We herein reported a case of spinal astrocytoma, where the patient underwent two surgeries and recurred three times. Adjuvant doublet chemotherapy following the first resection and chemoradiotherapy after the second excision both failed to thwart disease progression. A regimen containing palbociclib was therefore adopted upon identification of *CDK4* amplification using NGS-based genetic testing. The patient responded well and achieved complete response after 11 months of treatment.

This patient was indeed a rare case because he was triple-negative for *IDH* mutations, *TERT* promoter mutations and 1p/19q co-deletion, which is observed in only 7% of spinal astrocytoma patients according to a previous report, Wild-type *IDH1* or *IDH2* is associated with an increased risk of aggressive disease, and prognosis for triple-negative patients are even worse [10]. This is consistent with the fact that the patient in this case recurred in five months after the first surgery.

The cyclin D (*CCND1*)-CDK4/6-INK4-Rb pathway is a key regulator of the G1-S transition in the cell cycle. When activated by mitogenic signaling, *CCND1* binds with *CDK4/6* to form a complex which phosphorylates Rb and thereby releases E2F from the transcriptionally repressive Rb-E2F complex. E2F is thus free to promote transcription of genes required for cell cycle progression and DNA replication [5]. Amplification of the *CCND1*, *CDK4*, or *CDK6* genes or loss-of-function mutations in cyclin-dependent kinase inhibitor 2A (CDKN2A) are the primary mechanisms for overactivation of the CCND1-CDK4/6-INK4-Rb pathway [6]. It was previously reported that *CDK4* amplification occurs in 15% of malignant gliomas [10]. Palbociclib is the first-in-class CDK4/6 inhibitor and has been granted FDA approval for either first-line use in combination with an aromatase inhibitor (AI) in hormone receptor positive (HR+) human epidermal growth factor receptor 2 negative (HER2–) metastatic breast cancer (MBC) or in pretreated MBC patients in combination with fulvestran. Although it has not been approved yet to treat *CDK4-*altered solid tumors, palbociclib directly targets *CDK4* by binding to its ATP pocket. Moreover, it was previously shown that palbociclib monotherapy produced a favorable PFS rate in liposarcoma [7]. There are also multiple ongoing trials (NCT03454919, NCT03242382, NCT01037790, and NCT02806648) investigating efficacy and safety of palbociclib in multiple malignancies with CDK4 overexpression (www.clinicaltrials.gov). Palbociclib was therefore started upon resistance to treatment with bevacizumab, irinotecan, and temozolomide, with the patient’s consent. The remarkable response of *CDK4-*amplified CNS tumor to palbociclib-based multi-modality therapy as observed in the present case was not seen in another study attempting to match high grade glioma patients with targeted agents based on genomic sequencing results. In that study, seven out of 43 (16.3%) cases carried *CDK4* amplification, and palbociclib failed to elicit any response in a 65-year old patient following 2-months of treatment [11]. There are two possible explanations for this discrepancy: first of all, the 65-year old patient in that study had more advanced disease with a low Karnofsky score (KPS) at the time of palbociclib treatment; secondly, although palbociclib was able to prolong survival in mouse models of glioma, it has low blood-brain barrier (BBB) permeability as indicated by an unbound brain-to-plasma partition coefficient (Kp, uu) of 0.01 five minutes following intravenous administration in xenografts [12–14]. In our case, the patient underwent gamma knife radiosurgery before palbociclib treatment which might have improved the intake of palbociclib, given multiple lines of evidence showing the destruction of BBB after radiotherapy [15].

Administration of bevacizumab, irinotecan, and temozolomide after radiation was not effective for disease control in our case. It was not surprising since the addition of bevacizumab to temozolomide only had palliative effects on patients’ outcomes, and the value of chemotherapy and bevacizumab in spinal cord tumors is still inconclusive [16]. Another anti-angiogenic agent apatinib, however, was effective in patients with refractory high-grade gliomas when administered alongside chemotherapeutic agents such as temozolomide and irinotecan [8, 17]. This could have, in part, contributed to the exceptional response of the patient to the palbociclib-apatinib-temozolomide regimen despite multiple lines of previous treatment. The divergent effects of apatinib and bevacizumab could be explained by the fact that apatinib targets the intracellular domain of vascular endothelial growth factor receptor 2 (VEGFR-2) and hence induces tumor cell apoptosis by inhibiting autocrine VEGF signaling [18, 19]. Moreover, apatinib could reverse ATP-binding cassette (ABC) transporter-mediated multidrug resistance and enhance the efficacy of chemotherapy [20].

This case is of particular interest to us because it is the first case of spinal cord tumor ever reported to demonstrate an association between *CDK4* amplification and response to palbociclib-based combination therapy even after multiple recurrences. The success with this case corroborates the notion that both comprehensive genomic profiling and a multi-modality treatment strategy are critical for personalized therapy of rare cancer types.

## Supplementary information

**Additional file 1: Table S1.** Markers examined using IHC. **Table S2.** A list of the 381 genes included in the NGS panel

## Data Availability

The datasets generated and analyzed during this study are not publicly available but are available from the corresponding author on reasonable request.

## Abbreviations

ABC: ATP-binding cassette
AI: Aromatase inhibitor
BBB: Blood-brain barrier
CDK4: Cyclin dependent kinase 4
CNS: Central nervous system
CCND1: Cyclin D
CT: Computed tomography
FRS2: Fibroblast growth factor receptor substrate 2;
GLI1: GLI family zinc finger 1
HR+: Hormone receptor positive.
H3F3A: H3.3 histone A
HIST1H3B: Histone cluster 1, H3b
IDH: Isocitrate dehydrogenase
KPS: Karnofsky score
MBC: Metastatic breast cancer
MDM2: MDM2 proto-oncogene
MRI: Magnetic resonance imaging
NCCN: National Comprehensive Cancer Network
NGS: Next-generation sequencing
PFS: Progression free survival
PTPN11: Protein tyrosine phosphatase non-receptor type 11
PET-CT: Positron emission tomography-computed tomography
SCA: Spinal cord astrocytoma
VEGFR-2: Vascular endothelial growth factor receptor 2
WEE1: WEE1 G2 checkpoint kinase
