# Supplementary material for: A refractory case of CDK4-amplified spinal astrocytoma achieving complete response upon treatment with a Palbociclib-based regimen:a case report
Source: BMC Cancer. 2020 Jul 8;20:630. doi: 10.1186/s12885-020-07061-3 (PMC7346338; doi:10.1186/s12885-020-07061-3)
Supplement: Supplementary file 1 — Additional file 1: Table S1. Markers examined using IHC. Table S2. A list of the 381 genes included in the NGS panel [file 12885_2020_7061_MOESM1_ESM.docx]

| **Marker** | **Significance** |
| --- | --- |
| Vimentin | The major intermediate filament present in nonmuscle cells; a sarcoma tumor marker to confirm mesenchymal origin of some tumors. |
| GFAP | An intermediate filament protein expressed by various cell types of the central nervous system; an index of gliosis or a relatively slow-developing correlate of neural damage. |
| S-100 | The most widely used marker for peripheral nerve sheath tumors. |
| Syn | A protein highly expressed in the advanced stages of many types of cancers; a marker of stimulated proliferation, metastasis and drug resistance |
| Ki-67 | A key marker associated with aggressively proliferating cancer cells |
| p53 | A marker for differentiating malignant conditions, which are often p53+ (carcinoma in situ of urothelium and other sites, invasive carcinoma) from reactive and metaplastic conditions which are usually p53-. |
| CgA | A marker used to identify a variety of neuroendocrine tumours. |
| EMA | Useful in identifying epithelial derivation of poorly differentiated malignant tumors. |

**Supplemental Table 1 Markers examined using IHC**

**Supplemental Table 2 A list of the 381 genes included in the NGS panel**

| ABL1 | BRAF | CSK | FANCC | GLI3 | KLHL6 | NEK11 | PPP2R1A | SMAD2 | TP53 |
| --- | --- | --- | --- | --- | --- | --- | --- | --- | --- |
| ABL2 | BRCA1 | CSNK1A1 | FANCD2 | GNA11 | KMT2A | NF1 | PRDM1 | SMAD3 | TPMT |
| ACVR1B | BRCA2 | CTCF | FANCE | GNA13 | KMT2C | NF2 | PREX2 | SMAD4 | TSC1 |
| ACVR2A | BRD4 | CTNNA1 | FANCF | GNAQ | KMT2D | NFE2L2 | PRKAR1A | SMARCA2 | TSC2 |
| ADAM29 | BRIP1 | CTNNB1 | FANCG | GNAS | KRAS | NFKBIA | PRKCI | SMARCA4 | TSHR |
| ADGRA2 | BTG1 | CUL3 | FANCL | GRIN2A | LCK | NKX2-1 | PRKDC | SMARCB1 | TYK2 |
| AKT1 | BTK | CXCR4 | FAS | GRM3 | LIMK1 | NOTCH1 | PRSS8 | SMO | U2AF1 |
| AKT2 | C11orf30 | CYLD | FAT1 | GSK3B | LMO1 | NOTCH2 | PTCH1 | SNCAIP | UGT1A1 |
| AKT3 | CARD11 | CYP2C19 | FBXW7 | H3F3A | LRP1 | NOTCH3 | PTEN | SOCS1 | VEGFA |
| ALK | CBFB | CYP2D6 | FGF10 | HCK | LRP1B | NPM1 | PTK2 | SOX10 | VHL |
| AMER1 | CBL | DAXX | FGF14 | HGF | LYN | NRAS | PTK6 | SOX2 | WEE1 |
| APC | CCND1 | DDR1 | FGF19 | HNF1A | LZTR1 | NRG1 | PTPN11 | SOX9 | WEE2 |
| AR | CCND2 | DDR2 | FGF23 | HRAS | MAGI2 | NRG3 | QKI | SPEN | WISP3 |
| ARAF | CCND3 | DICER1 | FGF3 | HSD3B1 | MAP2K1 | NSD1 | RAC1 | SPOP | WT1 |
| ARFRP1 | CCNE1 | DNMT3A | FGF4 | HSP90AA1 | MAP2K2 | NTRK1 | RAD50 | SPTA1 | XIAP |
| ARID1A | CD274 | DOT1L | FGF6 | IDH1 | MAP2K4 | NTRK2 | RAD51 | SRC | XPO1 |
| ARID1B | CD79A | DPYD | FGFR1 | IDH2 | MAP3K1 | NTRK3 | RAF1 | SRMS | YES1 |
| ARID2 | CD79B | EGF | FGFR2 | IGF1R | MAP4K5 | NUP93 | RANBP2 | STAG2 | ZBTB2 |
| ASXL1 | CDC73 | EGFR | FGFR3 | IGF2 | MCL1 | PAK3 | RARA | STAT3 | ZNF217 |
| ATM | CDH1 | EP300 | FGFR4 | IKBKE | MDM2 | PALB2 | RB1 | STAT4 | ZNF703 |
| ATR | CDK12 | EPHA2 | FGR | IKZF1 | MDM4 | PARK2 | RBM10 | STK11 | ZNF750 |
| ATRX | CDK4 | EPHA3 | FH | IL7R | MED12 | PAX5 | RET | STK24 |  |
| AURKA | CDK6 | EPHA5 | FLCN | INHBA | MEF2B | PBRM1 | RICTOR | SUFU |  |
| AURKB | CDK8 | EPHA7 | FLT1 | INPP4B | MEN1 | PDCD1LG2 | RIT1 | SYK |  |
| AXIN1 | CDKN1A | EPHB1 | FLT3 | IRF2 | MET | PDGFRA | RNF43 | TAF1 |  |
| AXL | CDKN1B | ERBB2 | FLT4 | IRF4 | MITF | PDGFRB | ROCK1 | TBX3 |  |
| BAP1 | CDKN2A | ERBB3 | FOXL2 | IRS2 | MLH1 | PDK1 | ROCK2 | TCF7L2 |  |
| BARD1 | CDKN2B | ERBB4 | FOXP1 | ITK | MPL | PIK3C2B | ROS1 | TEK |  |
| BCL2 | CDKN2C | ERCC1 | FRS2 | JAK1 | MRE11A | PIK3CA | RPTOR | TERT |  |
| BCL2L1 | CEBPA | ERG | FUBP1 | JAK2 | MS4A1 | PIK3CB | RUNX1 | TET2 |  |
| BIM | CHD2 | ERRFI1 | FYN | JAK3 | MSH2 | PIK3CD | RUNX1T1 | TGFBR1 |  |
| BCL2L2 | CHD4 | ESR1 | GABRA6 | JUN | MSH6 | PIK3CG | RXRA | TGFBR2 |  |
| BCL6 | CHEK1 | ETV1 | GATA1 | KAT6A | MST1R | PIK3R1 | SDHA | TIE1 |  |
| BCOR | CHEK2 | ETV4 | GATA2 | KDM5A | MTOR | PIK3R2 | SDHB | TMPRSS2 |  |
| BCORL1 | CIC | ETV5 | GATA3 | KDM5C | MUTYH | PKD2 | SDHC | TNFAIP3 |  |
| BCR | CRBN | ETV6 | GATA4 | KDM6A | MYB | PLA2G1B | SDHD | TNFRSF14 |  |
| BIRC5 | CREBBP | EZH2 | GATA6 | KDR | MYC | PLCG2 | SETD2 | TNFSF11 |  |
| BLK | CRKL | FAM135B | GID4 | KEAP1 | MYCL | PMS2 | SF3B1 | TNK2 |  |
| BLM | CRLF2 | FAM46C | GLI1 | KEL | MYCN | POLD1 | SIK1 | TOP1 |  |
| BMX | CSF1R | FANCA | GLI2 | KIT | MYD88 | POLE | SLIT2 | TOP2A |  |
